# Foot and Ankle Somatosensory Deficits Affect Balance and Motor Function in Children With Cerebral Palsy

**DOI:** 10.3389/fnhum.2020.00045

**Published:** 2020-02-26

**Authors:** Anastasia Zarkou, Samuel C. K. Lee, Laura A. Prosser, John J. Jeka

**Affiliations:** ^1^Spinal Cord Injury Research Laboratory, Crawford Research Institute, Shepherd Center, Atlanta, GA, United States; ^2^Department of Physical Therapy and Interdisciplinary Graduate Program in Biomechanics and Movement Science, University of Delaware, Newark, DE, United States; ^3^Research Department, Shriners Hospital for Children, Philadelphia, PA, United States; ^4^Department of Pediatrics, University of Pennsylvania & The Children’s Hospital of Philadelphia, Philadelphia, PA, United States; ^5^Department of Kinesiology and Applied Physiology, University of Delaware, Newark, DE, United States

**Keywords:** cerebral palsy, somatosensation, sensory function, balance, postural control, motor function

## Abstract

Sensory dysfunction is prevalent in cerebral palsy (CP). Evidence suggests that sensory deficits can contribute to manual ability impairments in children with CP, yet it is still unclear how they contribute to balance and motor performance. Therefore, the objective of this study was to investigate the relationship between lower extremity (LE) somatosensation and functional performance in children with CP. Ten participants with spastic diplegia (Gross Motor Function Classification Scale: I-III) and who were able to stand independently completed the study. Threshold of light touch pressure, two-point discriminatory ability of the plantar side of the foot, duration of cutaneous vibration sensation, and error in the joint position sense of the ankle were assessed to quantify somatosensory function. The balance was tested by the Balance Evaluation System Test (BESTest) and postural sway measures during a standing task. Motor performance was evaluated by using a battery of clinical assessments: (1) Gross Motor Function Measure (GMFM-66-IS) to test gross motor ability; (2) spatiotemporal gait characteristics (velocity, step length) to evaluate walking ability; (3) Timed Up and Go (TUG) and 6 Min Walk (6MWT) tests to assess functional mobility; and (4) an isokinetic dynamometer was used to test the Maximum Volitional Isometric Contraction (MVIC) of the plantar flexor muscles. The results showed that the light touch pressure measure was strongly associated only with the 6MWT. Vibration and two-point discrimination were strongly related to balance performance. Further, the vibration sensation of the first metatarsal head demonstrated a significantly strong relationship with motor performance as measured by GMFM-66-IS, spatiotemporal gait parameters, TUG, and ankle plantar flexors strength test. The joint position sense of the ankle was only related to one subdomain of the BESTest (Postural Responses). This study provides preliminary evidence that LE sensory deficits can possibly contribute to the pronounced balance and motor impairments in CP. The findings emphasize the importance of developing a thorough LE sensory test battery that can guide traditional treatment protocols toward a more holistic therapeutic approach by combining both motor and sensory rehabilitative strategies to improve motor function in CP.

## Introduction

Sensory inputs are crucial for the developing nervous system because they allow for the proper synaptic organization of the brain. In particular, somatosensory information is important for motor learning in the early stages of development and provides the foundation for acquiring more complex behavioral skills (Cascio, 2010; Maitre et al., 2012). Abnormal somatosensory processing has been associated with communication, motor, and social skill deficits in a range of neurodevelopmental disorders like cerebral palsy (CP; Cascio, 2010). Even though CP has been traditionally characterized as a developmental disorder of movement and posture, the reclassification of CP acknowledges coexistent sensory information and sensory processing deficits associated with this pathology (Rosenbaum et al., 2007).

Sensory deficits in CP have been primarily attributed to the injury of the immature brain and, secondarily, stem as a result of limited learning experience (Clayton et al., 2003; Rosenbaum et al., 2007) because motor impairments may not allow environmental exploration; a crucial element in development. Numerous imaging studies showed thalamocortical pathway disruption and aberrant somatosensory cortical activation in children with spastic CP (Burton et al., 2009; Kurz et al., 2014a,b, 2015; Papadelis et al., 2014, 2018), suggesting sensory processing dysfunction. Further, evidence demonstrated that the desynchronization of neuronal discharges in the somatosensory cortex has been related to the amount of error in ankle force performance (Kurz et al., 2014b), indicating that impaired feedback mechanisms can affect the skeletal musculature’s ability of persons with CP to adapt in a changing environment. Abnormal sensorimotor oscillatory activity during a knee extension task has shown that children with CP may have anticipatory feedforward control deficits, as their limited environmental exploration early in life does not allow them to develop appropriate internal models for a successful motor response (Kurz et al., 2014a). Altogether, the aforementioned findings suggest that sensory processing deficits associated with this pathology may lead to impaired motor planning and diminished postural control.

Clinical studies have reported somatosensory impairments in upper extremities (Cooper et al., 1995; Wingert et al., 2008, 2010; Auld et al., 2012a,b) affecting up to 90% of children with hemiplegia (Bleyenheuft and Gordon, 2013). Most of these studies showed tactile deficits that have been associated with poor unimanual and bimanual motor performance and inability to characterize an object by its properties (i.e., weight, texture, shape, etc.; Auld et al., 2012b). Additionally, impaired somatosensory integration has negatively influenced feedforward motor control mechanisms during precision grip tasks even in cases where only one hand was primarily affected as in unilateral CP (Bleyenheuft and Gordon, 2013). By using a fingertip force paradigm, Gordon et al. (1999) showed that children with hemiplegia presented anticipatory control deficits in the affected hand due to disrupted sensory information (Gordon and Duff, 1999). In a systematic review of the precision grip and sensory impairments in CP, it was concluded that the relationship between sensory dysfunction and prehension deficits needs to be delineated to improve the design of more focused and effective neurorehabilitation approaches for manual function (Bleyenheuft and Gordon, 2013).

Studies have also found that children with CP exhibit lower extremity (LE) somatosensation deficits (McLaughlin et al., 2005; Wingert et al., 2009). Specifically, impairments in pain (McLaughlin et al., 2005), position sense of the knee (McLaughlin et al., 2005) and hip (Wingert et al., 2009), and direction of scratch (McLaughlin et al., 2005) have been reported in spastic CP. Kurz et al. (2015) provided evidence on the relationship between somatosensory cortical activation and mobility as they showed that an abnormal cortical response to plantar tactile stimulation may have a negative impact on walking ability and plantar flexors’ strength in this population. Also, hip proprioception deficits in children with unilateral and bilateral CP have been linked to increased postural sway and decreased gait velocity, even when visual information was upregulated (Damiano et al., 2013). Overall, deficits in sensory information and processing contribute to motor impairments; however, for children with CP, the relationship between foot and ankle somatosensory function and balance performance is not clear.

The aim of the current study was to delineate the contribution of decreased plantar cutaneous feedback and inaccurate ankle proprioceptive input on balance control and motor performance in children with CP. We hypothesized that plantar cutaneous and ankle proprioception deficits would be related to impaired balance and motor function in this population. The findings shed light on how to design more effective sensory-oriented rehabilitative protocols in CP.

## Materials and Methods

### Participants

Ten ambulatory children with spastic diplegia, who were able to stand without any assistive device, were recruited from the outpatient CP clinic at Shriners Hospital for Children in Philadelphia, PA, USA. All participants were able to follow multiple-step commands to complete the somatosensory assessments and clinical measures. Children with a history of the selective dorsal rhizotomy, a score of 4 on the modified Ashworth scale, severe scoliosis (primary curve >40°), LE joint instability, and marked visual, hearing, and vestibular deficits were excluded from the study. Additional exclusion criteria were: LE orthopedic surgery or fracture in the year prior participation, botulinum toxin injections within the past 6 months, and pregnancy if the participant was female. The protocol was approved by the Western Institution Review Board (IRB) and the IRB of Temple University and the University of Delaware. Informed parental consent and child assent or consent forms in accordance with the Declaration of Helsinki were obtained prior to participation.

### Experimental Procedures

#### Somatosensory Function

All the children completed a comprehensive clinical evaluation to document their foot and ankle somatosensory function. *Light touch pressure* sensation was assessed by using the 6-item Monofilaments kit (Baseline^®^, White Plains, New York, NY, USA) at the first and fifth metatarsal heads and heel of the plantar side of each foot (Meyer et al., 2004; Citaker et al., 2011; Cruz-Almeida et al., 2014). The light touch pressure threshold was defined as the thinner monofilament value the participant correctly identified twice out of three trials for each application site. *Two-point discrimination* was assessed by using an aesthesiometer (Baseline^®^, White Plains, New York, NY, USA) on the forefoot and heel of the plantar side of each foot (Meyer et al., 2004; Citaker et al., 2011), and scored as the minimum distance in mm between two stimulus points (Meyer et al., 2004; Citaker et al., 2011; Auld et al., 2012a), which were correctly identified as distinct points twice out of three trials for each site. *Cutaneous vibration sensation* was evaluated by using a 128 Hz tuning fork (Rydel–Seiffer graduated tuning fork, Martin Tuttlingen, Germany) at the first metatarsal head and medial malleolus bilaterally (McLaughlin et al., 2005; Citaker et al., 2011). This is a reliable and valid clinical tool that is used to evaluate vibration perception impairments (Alanazy et al., 2018; Marcuzzi et al., 2019). The duration of the perceived vibration stimulus (average of three trials) for each site was recorded. For the ankle *joint position sense* assessment, the participant was instructed to actively reproduce, as accurately as possible, a target joint angle position for each leg. The magnitude of error between the performance and target joint angle was recorded to the nearest degree (average of three trials) for each ankle (Wingert et al., 2009; Damiano et al., 2013).

All the aforementioned testing procedures were performed in random order, without visual feedback, and the total testing duration was approximately 1 h. To determine an individual’s threshold for each somatosensory test (overall score) and each site of sensory stimulus application (site-specific score), the average of the combined left and right side scores were computed. In addition, the overall score for each somatosensory test was calculated by averaging the values of all the application sites for every somatosensory modality. Both overall and site-specific scores were used in the analyses.

#### Balance Performance

##### Postural Control

The Balance Evaluation Systems Test (BESTest) is a 36-item physical performance scale and was employed to assess balance in the following postural control domains: (1) Biomechanical Constraints; (2) Stability Limits/Verticality; (3) Anticipatory Postural Adjustments; (4) Postural Responses; (5) Sensory Orientation; and (6) Stability in Gait (Horak et al., 2009). Each item was assessed on a four-point scale and percentage scores were calculated for each domain with higher scores suggesting better balance performance. An overall BESTest score was also computed. The BESTest can discriminate postural control abilities in children with typical development with high reproducibility (Dewar et al., 2017) and has been also used previously in children with CP to evaluate balance after the completion of a treadmill training protocol (Kurz et al., 2011).

##### Standing Balance

Standing balance was assessed by postural sway measures (COP-based measures; Zarkou et al., 2018). Children stood barefoot on two force plates with their feet in a neutral position—the distance between heels was approximately 11% of each subject’s height and at a 14° degrees angle between each foot and the midline (Hwang et al., 2014). Tape traces of the feet on the force plates were used to ensure consistent positioning between trials. The children were instructed to stay as motionless and upright as possible and were asked to keep their gaze straight ahead at the eye level. The duration of each trial was 25 s for a total of two trials and the resting interval between trials depended on each participant’s comfort and fatigue level. Finally, an overhead harness system was used to prevent falls during each trial.

For kinetic assessment of balance, two AMTI force plates (OR6-7-1000, Advanced Mechanical Technology Inc., Watertown, MA, USA) were used. The force plate data were collected by using Vicon Nexus software (v1.8.5) at 100 Hz sampling rate and filtered with a fourth-order, zero-phase response, low-pass Butterworth filter with a cutoff frequency of 5 Hz (Prieto et al., 1996; Ross et al., 2013). Then, the resultant COP velocity (COPV) and 95% COP Confidence ellipse area (COPA) were computed (Zarkou et al., 2018) and used to investigate their relationship with LE light touch pressure, two-point discrimination, cutaneous vibration, and ankle joint position sense.

#### Motor Performance

##### Gross Motor Ability

The Gross Motor Function Measure Item Set (GMFM-66-IS), the abbreviated version of Gross Motor Function Measure 66 (GMFM-66), is a standardized instrument designed to measure the change in gross motor function in children with CP (Russell et al., 2010). For GMFM-66-IS, an algorithm of three decision items from GMFM-66 (items 23, 67, and 85) was used to define which of the four available item sets can be administered (Russell et al., 2010) to more accurately represent each child’s function level. It has been reported that there is no systematic difference between different item sets (Russell et al., 2010) with high levels of validity and reliability (ICC > 0.98; Brunton and Bartlett, 2011). For the purposes of this study, the item sets 3 (*n* = 39 items) and 4 (*n* = 22 items) were used since our participants had only mild mobility impairments (GMFCS I- III; they were able to stand without the assistive device). Each item was graded on a four-point scale ranging from 0 (does not initiate the required task) to 3 (completes the required task) and was scored by a physical therapist using GMAE software.

##### Walking Ability

Spatiotemporal characteristics of gait were evaluated while children walked on an instrumented walkway (GAITRite^®^, CIR Systems Inc., Franklin, NJ, USA). The GAITRite mat was positioned on the floor and children started walking 1.2 m before the beginning of the mat (acceleration walkway) and continued walking 1.2 m after reaching the end of the mat (deceleration walkway). The acceleration and deceleration walkways ensured that the children walked on a steady speed over the instrumented walkway. Subjects were tested in bare feet walking at their fast speed and without using any assistive device. Two to six trials were collected depending on children’s number of steps per trial (i.e., at least 16 steps). To qualify as a valid walking pass, trials had to include at least four consecutive footfalls on the instrumented walkway. The first four gait cycles for each side (right and left) were used for further analyses; thus, collecting a total of eight strides allowed for reliable estimation of gait parameters in children with CP (GMFCS I-III; Redekop et al., 2008). The following spatiotemporal parameters were collected: gait speed and step length normalized to height (Non-Dimensional approach; Stansfield et al., 2003). All values from the selected gait cycles were averaged for each variable of interest.

##### Functional Mobility

The Timed Up and Go (TUG) test quantifies functional mobility. Children rose from a seated position, walked 3 m, turned around, and walked back to the chair and sat down as quickly and safely as possible (Williams et al., 2005). The test was repeated three times and the average time was recorded. Participants performed the test barefoot without using an assistive device. The 6-Minute Walk Test (6MWT) assessed children’s walking aerobic capacity (Maher et al., 2008; Fitzgerald et al., 2016). Each subject was asked to ambulate around a fixed course as safely and quickly as possible. The distance that the individual was able to traverse in the allotted time was recorded. Only one child with CP needed to use a walker to complete the test.

##### Strength

The Maximum Volitional Isometric Contraction (MVIC) of triceps surae, bilaterally, was assessed by a computerized controlled dynamometer (KinCom II, Chattecx Corporation, Chattanooga, TN). Children were positioned in the dynamometer for triceps surae testing as previously described in the literature (Stackhouse et al., 2005). A total of three trials for each side were collected with a 3-min resting period between trials. During each trial, visual feedback and enthusiastic verbal encouragement were provided to children. The peak MVIC value was normalized with each subject’s body weight and then the left and right MVIC were averaged and used for subsequent analyses.

### Statistical Analyses

Due to the small sample size of this pilot study, median and interquartile ranges (IQR) were computed for the demographic characteristics and the sensory and motor function clinical assessments. Further, Spearman rank correlation coefficients were calculated to determine the relationships between somatosensory function and the respective clinical measures that assess balance and motor performance. According to Cohen’s standards, rho coefficients greater than 0.5 indicate strong relationships, 0.3–0.5 moderate relationships, and 0.1–0.3 weak relationships (Cohen, 1988). Statistical significance was set at *p* < 0.05. The SPSS (version 23; SPSS Inc., Chicago, IL, USA) statistical software was used for the analyses.

## Results

A total of 10 children with CP participated in this study. The median and IQR for age, height, and weight were 15.62 years (13.37–18.15), 165.8 cm (150.5–170), and 58.25 kg (38.25–74.23) respectively. The demographic characteristics of each individual are presented in Table 1.

**Table 1:** Participants’ demographic information. All children were diagnosed with spastic diplegic cerebral palsy (CP).

	Age	Sex	GMFCS	Height	Weight
1	9 years 1 month	M	I	142 cm	33 kg
2	17 years 8 months	M	II	182.5 cm	127 kg
3	18 years 0 months	M	I	158.5 cm	58.3 kg
4	15 years 7 months	M	III	164.6 cm	49.1 kg
5	13 years 1 month	M	I	146 cm	30.2 kg
6	18 years 6 months	M	II	170 cm	66 kg
7	18 years 6 months	M	I	170 cm	58.2 kg
8	15 years 2 months	M	III	169 cm	98.9 kg
9	15 years 6 months	M	III	167 cm	59.3 kg
10	13 years 5 months	F	II	152 cm	40 kg

*Abbreviations: GMFCS, Gross Motor Function Classification Scale*.

For the rest of the foot and ankle somatosensory tests and motor function clinical measures, the median and IQR values are presented in Table 2.

**Table 2:** Median and Interquartile Range (IQR) values for somatosensory and motor function assessments in children with CP.

Sensory assessments	Median (IQR)	Motor ability assessments	Median (IQR)
Light touch pressure (level)		Postural control (%)
1st Metatarsal	4.31 (4.14–4.61)	BESTest *Overall*	62.96 (40.28–83.10)
5th Metatarsal	4.31 (4.14–4.9)	BESTest 1 *Biomechanical Constraints*	56.67 (33.33–81.67)
Heel	4.38 (4.22–5.18)	BESTest 2 *Stability Limits/Verticality*	78.57 (63.10–85.71)
Overall	4.33 (4.14–4.79)	BESTest 3 *Anticipatory Postural Adjustments*	52.78 (48.61–91.67)
Two-point discrimination (mm)		BESTest 4 *Postural Responses*	30.55 (19.44–55.55)
Forefoot	17.5 (13.75–21.25)	BESTest 5 *Sensory Orientation*	76.67 (48.33–100.00)
Heel	17.5 (16.88–25.63)	BESTest 6 *Stability in Gait*	64.29 (28.57–95.24)
Overall	17.5 (16.56–21.56)	Balance Performance
Vibration (s)		COPA (cm^2^)	81.10 (15.49–109.39)
1st Metatarsal	15.5 (14.46–20.58)	COPV (cm/s)	5.55 (4.16–7.25)
Medial Malleolus	16.17 (11.09–19.25)	Gross Motor Ability (%)
Overall	16.79 (12.83–19.19)	GMFM-66-IS	75.00 (68.78–89.03)
Joint Position Sense (degrees)		Walking Ability (ND)
Ankle	4.5 (3.10–5.17)	Velocity	0.33 (0.26–0.36)
		Step Length	0.35 (0.31–0.37)
		Functional Ability
		TUG (s)	7.84 (5.93–12.10)
		6MWT (min)	467.72 (381.10–534.31)
		Strength
		MVIC (N/Kg)	4.69 (2.04–6.83)

*Abbreviation: BESTest, Balance Evaluation Systems Test; COPV, center of pressure resultant velocity; COPA, 95% eclipse area of center of pressure; GMFM-66-IS, Gross Motor Function Measure Item Set; ND, Non-Dimensional; TUG, Timed Up and Go; 6MWT, 6-Minute Walk Test; MVIC, Maximum Volitional Isometric Contraction*.

### Relationships Between Somatosensation and Balance

Spearman rho correlation coefficients, presented in Table 3, were computed to assess the relationship between somatosensory function (overall scores) and balance performance.

**Table 3:** Spearman’s rank correlations between the somatosensation thresholds and balance control scores in children with CP.

	Somatosensory ability measures			
	Light touch pressure	Two-point discrimination	Vibration	JPS
Postural control				
BESTest *overall*	0.00	−0.64*	−0.31	−0.28
BESTest 1 *Biomechanical Constraints*	0.01	−0.62*	−0.12	−0.19
BESTest 2 *Stability Limits/Verticality*	0.24	−0.83**	−0.56*	−0.34
BESTest 3 *Anticipatory Postural Adjustments*	−0.25	−0.48	−0.39	0.00
BESTest 4 *Postural Responses*	0.35	−0.57*	−0.26	−0.70*
BESTest 5 *Sensory Orientation*	0.06	−0.58*	−0.14	−0.52
BESTest 6 *Stability in Gait*	−0.14	−0.69*	−0.52	−0.11
Balance Performance				
COPA	−0.31	0.86**	0.73**	0.23
COPV	0.01	0.45	0.69*	−0.09

Asterisks indicate significant relationships (*p < 0.05; **p < 0.01). Abbreviations: JPS, Joint Position Sense; BESTest, Balance Evaluation Systems Test; COPV, the center of pressure resultant velocity; COPA, 95% eclipse area of the center of pressure.

**Figure 1:**
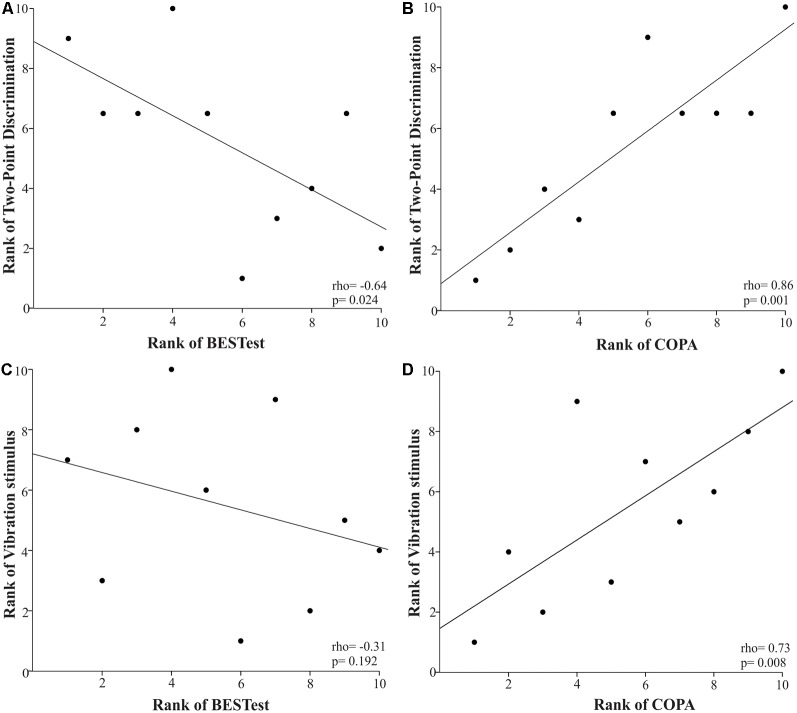
Scatter plots of the relationships between two-point discrimination **(A,B)** and vibration **(C,D)** senses and balance ability using Spearman’s rank correlations in the children with cerebral palsy (CP). Each data point reflects a participant.

Two-point discrimination was strongly related with the BESTest score in all subdomains (rho = −0.57 to −0.83, *p* < 0.05)—except for the *Anticipatory Postural Adjustments* subdomain—and the COPA (rho = 0.86, *p* = 0.001). A strong relationship was also revealed between vibration sensation and the *Stability Limits/Verticality* subdomain of BESTest (rho = −0.56, *p* = 0.048) and COP measures (COPV: rho = 0.69, *p* = 0.014; COPA: rho = 0.73, *p* = 0.008). Scatterplots partially summarize these results (Figure 1).

**Figure 2:**
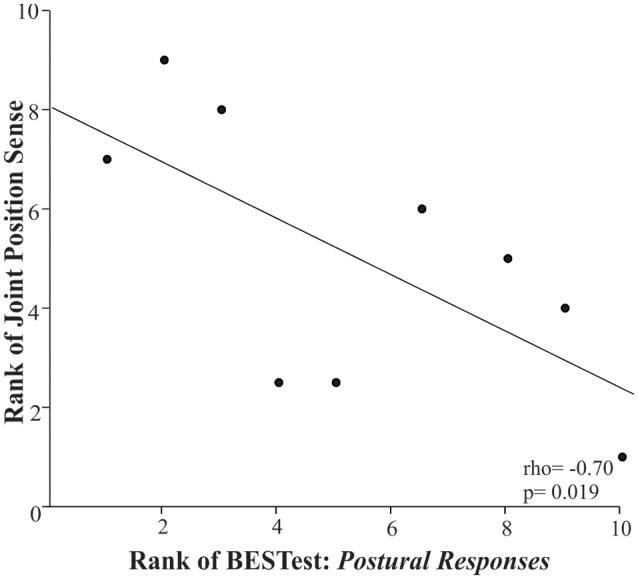
Scatter plot of the rank of the Balance Evaluation System Test (BESTest) score in the 4th Subdomain (postural responses) and the rank of the ankle joint position sense in the children with CP. Each data point reflects a participant.

Ankle joint position sense was significantly associated with the *Postural Responses* subdomain of BESTest (Figure 2: rho = −0.70, *p* = 0.019). For all the above relationships, the rho coefficients’ negative value indicated that the higher the somatosensory assessment thresholds indicating greater impairment, the lower children’s score in the BESTest; whereas, the positive value suggested that the higher the somatosensory thresholds the larger the postural sway measures during the standing balance test.

Spearman rho correlation coefficients were also computed to characterize the relationships between the site-specific scores of the somatosensory tests and the balance clinical measures (Table 4). In particular, there was a negative correlation between the two-point discrimination in the forefoot area with two of the subdomains of the BESTest (Stability Limits/Verticality: rho = −0.68, *p* = 0.015; Stability in Gait: rho = −0.58, *p* = 0.04). Similarly, two-point discrimination in the heel area was strongly related to three of the subdomains of BESTest (rho = −0.62 to −0.65, *p* < 0.05). Vibration sensation in the first metatarsal site demonstrated a strong negative relationship with the overall BESTest score (rho = −0.60, *p* = 0.033) and the score in Stability Limits/Verticality, Postural Responses, and Stability in Gait subdomains of BESTest (rho = −0.62 to −0.70, *p* < 0.05). These correlations suggested that the higher the two-point discrimination thresholds and the longer the vibration stimulus was perceived the poorer that participants performed in the BESTest, showing impaired postural control in these children (Table 4).

**Table 4:** Spearman’s rank correlations between two-point discrimination and vibration senses, at different application sites, and balance ability measures in children with CP.

	Two-point discrimination		Vibration	
	Forefoot	Heel	1st Metatarsal	Medial Malleolus
Postural control					
BESTest *Overall*	−0.45	−0.54	−0.60*	−0.05
BESTest 1 *Biomechanical Constraints*	−0.29	−0.62*	−0.54	0.13
BESTest 2 *Stability Limits/Verticality*	−0.68*	−0.65*	−0.69*	−0.15
BESTest 3 *Anticipatory Postural Adjustments*	−0.35	−0.29	−0.54	−0.22
BESTest 4 *Postural Responses*	−0.33	−0.64*	−0.70*	0.07
BESTest 5 *Sensory Orientation*	−0.31	−0.53	−0.51	0.21
BESTest 6 *Stability in Gait*	−0.58*	−0.47	−0.62*	−0.24
Balance Performance				
COPA	0.72**	0.74**	0.77**	0.46
COPV	0.65*	0.36	0.33	0.77**

Asterisks indicate significant relationships (*p < 0.05; **p < 0.01). Abbreviations: BESTest, Balance Evaluation Systems Test; COPV, the center of pressure resultant velocity; COPA, 95% eclipse area of the center of pressure.

Two-point discrimination in the forefoot and heel sites and cutaneous vibration sensation in the first metatarsal site showed a strong positive relationship with the COPA (rho = 0.72–0.77, *p* < 0.01). Additionally, increased two-point discrimination thresholds and longer vibration perception in the forefoot and medial malleolus areas, respectively, were significantly associated with the increased velocity of COP sway (rho = 0.65, *p* = 0.02 and rho = 0.77, *p* = 0.004). Finally, none to weak relationships were found between site-specific scores for light touch pressure and the balance performance measures.

### Relationships Between Somatosensation and Motor Function

**Figure 3:**
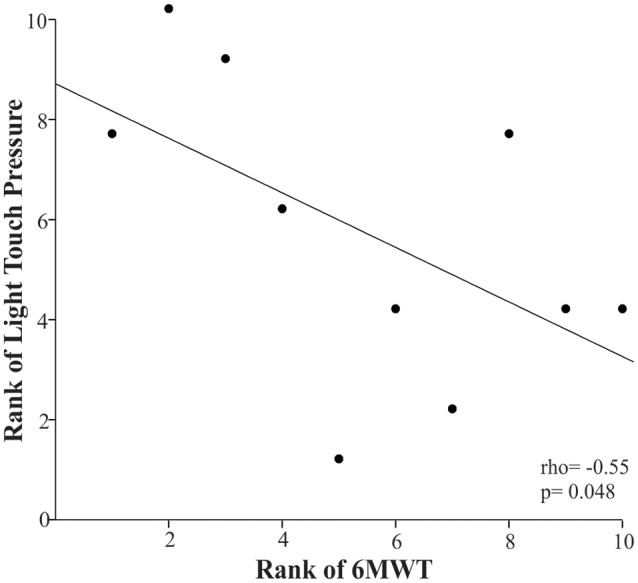
Scatter plot of the rank of the 6-Minute Walk Test (6MWT) and the rank of the light touch pressure in the children with CP. Each data point reflects a participant.

Children with higher light touch pressure thresholds in their plantar side of the foot (overall score) were more likely to cover a shorter distance during the 6MWT, as indicated by the negative rho coefficient of −0.55 (*p* = 0.048; Figure 3).

**Figure 4:**
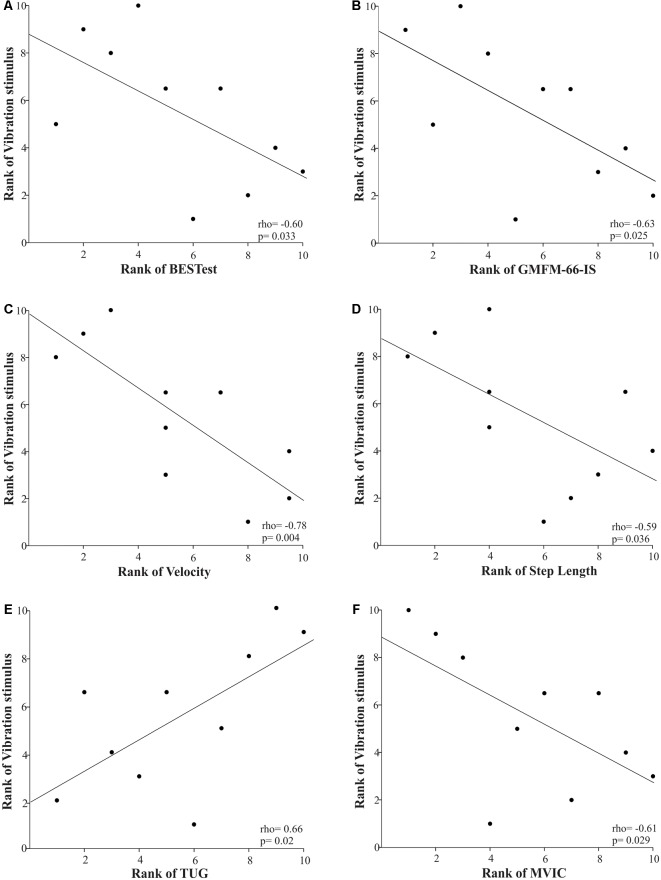
Scatter plots of the Spearman’s rank correlations between the rank of the vibration stimulus when applied in the first metatarsal area and the rank of motor performance variables [i.e., postural control **(A)**, gross motor function **(B)**, gait velocity **(C)**, step length **(D)**, functional mobility **(E)**, and plantar flexors’ strength **(F)**] in children with CP. Each data point reflects a participant.

Furthermore, only the cutaneous vibration sensation of the first metatarsal head demonstrated a significantly strong relationship with motor performance as measured by GMFM-66-IS, spatiotemporal gait parameters, TUG, and plantar flexor strength (Figure 4). More specifically, the longer the children were able to perceive the vibration stimulus in the first metatarsal area the more likely they were to have limitations in gross motor function (rho = −0.63, *p* = 0.025), and walking ability (gait velocity: rho = −0.78, *p* = 0.004; step length: rho = −0.59, *p* = 0.036), functionality (TUG: rho = 0.66, *p* = 0.02), and plantar flexors’ strength (rho = −0.61, *p* = 0.029). The rest of the somatosensory site-specific scores were weakly to moderately associated with motor function tests, and these relationships were not statistically significant.

## Discussion

This study investigated the relationship between foot and ankle somatosensory and motor function in children with spastic diplegic CP. Our results demonstrated that foot and ankle somatosensation is strongly related to standing balance and motor performance; thus, supporting the notion that plantar cutaneous and ankle proprioceptive deficits may contribute to the postural control and mobility impairments in this population. These clinical findings emphasized the importance of developing a thorough LE sensory test battery that can identify subject-specific sensory deficits and, therefore, guide traditional treatment protocols toward a more comprehensive therapeutic approach by combining motor and sensory rehabilitative strategies to improve motor function in CP. Also, our findings are particularly noteworthy in light of existing evidence indicating that enhancing sensory inputs through stochastic resonance stimulation applications can improve balance in children with CP (Zarkou et al., 2018).

### Relationship Between Somatosensation and Balance

Flexible postural control and motor planning require organizing and integrating visual, vestibular, and somatosensory inputs to efficiently coordinate motor actions (Shumway-Cook and Woollacott, 2012). Research indicates that individuals with CP may depend primarily on feedback from their visual and vestibular systems in environments that challenge balance compared to individuals without CP (Yu et al., 2019). Impairments in at least one of the aforementioned sensory systems could be a contributing factor in the poor balance control exhibited by children with CP. Postural control deficits in this population have been attributed to biomechanical changes in postural alignment as well as to central nervous system (CNS) sensory processing impairments (Papadelis et al., 2014; Kurz et al., 2015; Pavão et al., 2015). Our results showed that LE somatosensory function is strongly related to balance performance in CP and, therefore, impairments in the plantar cutaneous and ankle proprioceptive function may partially contribute to balance deficits.

Among the tested somatosensory modalities, two-point discrimination in the plantar side of the foot was significantly associated with all but one of the subdomains of BESTest and the area of COP sway during quiet stance. Specifically, the larger the distance between the two applied stimuli that children perceived as distinct, the poorer they performed in five different underlying systems that contributed to postural control, hence suggesting generalized balance problems in CP. When investigating the site-specific scores for two-point discrimination, we found that both the forefoot and heel areas contributed to the observed poor balance performance. These findings indicated that limited spatial and temporal tactile information from the anterior and posterior supporting zones of the foot (i.e., forefoot and heel areas; Kavounoudias et al., 1998) may result in inability to trigger the appropriate compensatory responses to maintain a stable upright stance in CP.

Vibration sensation in the first metatarsal area showed significant relationships with three subcategories of BESTest and the area of COP sway. These findings suggested that when children were able to perceive vibration sensation for a longer period of time, they showed decreased functional stability limits, impaired compensatory postural responses, and dynamic and static stability deficits. Previous research in vibration sensation reported that children with CP were not able to properly identify a vibration stimulus in their LE (McLaughlin et al., 2005). Further, we showed that children with CP, although they did not perform significantly different compared to controls, they perceived the vibration stimulus for a longer period (Zarkou, 2017). Conversely, for individuals with multiple sclerosis (Citaker et al., 2011) the duration of the perceived vibration is shorter compared to healthy adults and this has been attributed to spinal dorsal column abnormalities associated with this pathology (Zackowski et al., 2009). Temlett (2009) showed that the duration of the vibration sensation also declines with age due to nerve fibers degeneration and deterioration of Pacinian corpuscles, which are the primary mechanoreceptors of cutaneous vibration sensation. Evidence suggests that vibrotactile stimulation at the foot activates Pacinian corpuscles, but can also modulate ankle joint proprioception, thus indicating an interplay between tactile and proprioceptive inputs (Mildren and Bent, 2016) that both contribute to postural control (Kavounoudias et al., 2001). In this study, the recorded longer period of vibration sensation in CP may have indicated aberrant and prolonged processing and integration of the afferent vibratory input by the CNS that resulted in impaired balance control. This corroborates brain imaging findings proposing that sensory processing deficits contribute to the motor planning and execution impairments in spastic diplegia (Burton et al., 2009; Kurz et al., 2014a,b; Kurz et al., 2015).

Ankle joint position sense errors were significantly related to the *Postural Responses* subdomain of BESTest. In particular, for this category’s balance tasks, children were required to regain their equilibrium with and without taking a step following perturbations in different directions (i.e., forward, backward, or lateral) induced by the examiner’s hands (Horak et al., 2009). Children with CP were unable to elicit an appropriate postural response to unexpected perturbations, receiving a median group score of 30.55 out of the maximum 100. The lower they scored in this subdomain, they presented larger errors in reproducing the target ankle position during the joint position sense test. These findings potentially demonstrate that ankle proprioceptive deficits did not allow for proper sensory feedback during the execution of the motor response and, therefore, children with CP were unable to regain equilibrium during a challenging balance task. Similarly, Damiano et al. (2013) reported that increased hip proprioception errors were significantly related to increased postural sway during quiet stance and decreased gait velocity in CP. Altogether, the findings suggested that evaluation of proprioception should be incorporated into LE sensory battery tests, especially in light of evidence that proprioceptive deficits can be exacerbated by the loss of plantar cutaneous inputs affecting balance stability (Meyer et al., 2004).

### Relationship Between Somatosensation and Motor function

In our previous work, we showed that light touch pressure thresholds significantly increased in children with CP compared to their age-matched typically developing peers (Zarkou, 2017). Although higher light touch pressure thresholds have been associated with poor balance performance in older adults (Cruz-Almeida et al., 2014), individuals with multiple sclerosis (Citaker et al., 2011), and peripheral neuropathy (Perkins et al., 2001; Kars et al., 2009), this study demonstrated that the only significant relationship in children with CP was between light touch pressure and the 6MWT, yet it was not associated with balance measures. Specifically, higher light touch pressure thresholds were significantly related to shorter distances covered over a 6 min period. A possible explanation is that during dynamic activities like gait, in which the loading response may be equivalent to several times the bodyweight of the individual, the plantar mechanoreceptors’ thresholds are more likely to be reached compared to simpler balance tasks that involve lower levels of plantar pressure like the ones that occur during postural shifts to maintain standing balance (Cruz-Almeida et al., 2014). Therefore, the impact of the plantar light touch pressure deficits on postural stability in CP may be more evident during a prolonged walking task, like the 6MWT.

Interestingly, the cutaneous vibration sensation at the first metatarsal head was the only sensory modality that was significantly related to the majority of the clinical motor assessments. In particular, longer duration of the vibration perception was significantly related to impaired gross motor and walking function, functional mobility, and plantar flexors’ strength. These findings implied that vibratory inputs from the first metatarsal head, as provided by the stimulation of Pacinian corpuscles that are located at both the subcutaneous tissue, bony periosteum, and joint ligaments (Temlett, 2009), are crucial for static and dynamic postural control. Moreover, our findings corroborate previous work reporting that decreased sensory inputs from the first metatarsal head area have been associated with decreased score in the Berg Balance Scale and walking speed in older adults (Cruz-Almeida et al., 2014). Hence, future studies should focus on delineating the reweighting of LE somatosensory cues—especially from the first metatarsal head area- and how it affects motor function not only during static but also dynamic and prolonged activities.

This work highlighted the strong relationship between somatosensory function and variables of balance and motor performance in CP even with small sample size. These observations may imply that somatosensory dysfunction is highly pervasive in children with CP, however, we urge caution in interpreting these results because of the small sample size. Furthermore, we acknowledge the fact that musculoskeletal deficits, along with poor somatosensory ability, can contribute to the noted motor impairments witnessed in children with CP as this pathology is multifactorial. Finally, over the course of the past decade, neuroimaging evidence has supported the existence of somatosensory processing deficits and abnormal sensorimotor connectivity in this population (Burton et al., 2009; Kurz et al., 2014a,b, 2015; Papadelis et al., 2014, 2018), however, there is limited research on the clinically detectable LE somatosensory impairments. Combining brain-imaging techniques with our clinical assessment methods may have further strengthened the results of this study.

## Conclusion

Somatosensory system is essential to motor control by providing information for the formulation of the appropriate feedforward anticipatory strategy and for the regulation of the feedback mechanism, which allows the correction of performance errors during the execution of a motor plan (Ghez, 1991; Schmidt and Lee, 2011); therefore, impairments in this system may impact motor behavior. In support of prior imaging work (Burton et al., 2009; Kurz et al., 2014a,b, 2015; Papadelis et al., 2014, 2018), our clinical findings suggested that sensory processing dysfunction is partially contributed to the motor planning and execution impairments that affect postural control and motor function in CP. Specifically, we provided evidence that somatosensory deficits in the LEs, especially two-point discrimination and cutaneous vibration sensation, appear to strongly influence balance and motor performance in children with spastic diplegia. Therefore, addressing the reported somatosensory impairments may contribute to postural stability and functional mobility improvements in this population.

Our research proposed that using a simple battery of clinical tests to assess somatosensation allows for the identification of tactile and proprioceptive deficits, and thus provides important information for clinical care in CP. Further research is required to investigate the minimum necessary number of somatosensory assessments that should be included in the clinical practice. A short screening tool that includes modality and site-specific tests besides being administered in a timely manner can potentially identify motor function declines in CP. In addition, it can guide traditional treatment protocols toward a more holistic therapeutic approach by combining motor and sensory rehabilitative strategies to improve overall functionality and quality of life in CP.

## Data Availability Statement

The raw data supporting the conclusions of this article will be made available by the authors, without undue reservation, to any qualified researcher.

## Ethics Statement

The studies involving human participants were reviewed and approved by Western Institution Review Board (IRB), Temple University IRB, and University of Delaware IRB. Written informed consent to participate in this study was provided by the participants’ legal guardian/next of kin. Child assents were also obtained prior participation.

## Author Contributions

All authors contributed to the design of the study. AZ and LP ran the experiments. AZ analyzed the data, interpreted the results, drafted the manuscript and submitted the manuscript. SL, LP, and JJ provided thorough feedback and revised the manuscript. All authors approved the final manuscript.

## Conflict of Interest

The authors declare that the research was conducted in the absence of any commercial or financial relationships that could be construed as a potential conflict of interest.
